# Efficacy and Safety of a Digital Tapering Intervention for Patients Prescribed Opioids After Surgery: Protocol for a Prospective Exploratory Cohort Study

**DOI:** 10.2196/72317

**Published:** 2025-09-29

**Authors:** Bergdís Elsa Hjaltadottir, Anna Bryndís Blöndal, Prescriby LLC Team, Orthopedics Department Group, SÁÁ group

**Affiliations:** 1 Prescriby LCC Reykjavík Iceland; 2 Faculty of Pharmaceutical Sciences University of Iceland Reykjavik Iceland; 3 See Acknowledgments

**Keywords:** medication adherence, medication therapy management, pain, pain management, general surgery, arthroplasty, replacement, knee, arthroplasty, replacement, hip, analgesics, opioid, oxycodone, drug tapering, deprescriptions

## Abstract

**Background:**

More than 300 million surgical procedures are performed worldwide each year, and opioids remain a primary approach for managing acute postoperative pain. Studies have demonstrated that a significant number of patients do not discontinue opioid treatment and continue to use opioids for months or even years after surgery. Tapering and management of prescription opioids is a well-known practice and is a part of the current clinical guidelines on safe prescribing. Every patient should receive thorough monitoring, education, and a tapering plan when prescribed opioids or receiving refills after a prolonged treatment. There are challenges associated with tapering, including close follow-up, patient education, clinician time, and withdrawal safety. The evolution of smartphone app use for follow-up has shown promising results in some fields of medicine, and patients are increasingly interested in this approach.

**Objective:**

The objective of this study is to investigate the efficacy and safety of Prescriby services, comprising clinician management augmented with digital support, as a tapering intervention in patients after knee or hip replacement surgery.

**Methods:**

Efficacy will be measured in tapers successfully completed, doses successfully lowered during tapering, number of active users, satisfaction with the intervention, and patients successfully remaining off opioid medication at 6 and 12 months after the intervention. Participant safety will be monitored by assessing adverse effects during tapering using the numeric pain rating scale to assess the severity of pain. Participants are recruited via referrals from orthopedic clinics and orthopedic departments in the hospital after surgery during the 6-month study period to the Prescriby clinic, where they will receive a personalized tapering treatment and follow-up with a clinical pharmacist. Despite the existence of numerous clinical guidelines on tapering off dependence-inducing medications, there is limited knowledge of the outcomes of such tapering.

**Results:**

This study received funding in February 2024. Data collection for secondary outcomes started in November 2024 and ended in July 2025. Data collection for primary outcomes will start in June 2026 and finish in July 2026. As of manuscript submission in August 2025, 75 patients had been recruited into the study. Analysis of secondary data started in August 2025, and results will be published in the fourth quarter of 2025. Analysis of primary data will start in July 2026, and results will be published in the fourth quarter of 2026.

**Conclusions:**

We anticipate that persistent opioid use in standard treatment could be somewhere in the range of 10% to 15%. We hypothesize that the intervention group will have a lower rate of persistent opioid use. With regard to the secondary outcomes, we estimate that more than 85% of the participants in intervention group will be able to taper completely off their opioid medication and anticipate a high degree of satisfaction with the intervention.

**International Registered Report Identifier (IRRID):**

DERR1-10.2196/72317

## Introduction

### Opioid Epidemic

The global increase in opioid use over the past decade has raised concerns, contributing to what is commonly referred to as the “opioid crisis” [1]. The opioid epidemic has been a long-standing battle. In the late 1990s, pharmaceutical companies advised the medical community that patients would not become addicted to opioid pain relievers. This led to increased prescription of opioids and widespread misuse of both prescription and illicit opioids before it became clear that these medications could indeed be highly addictive. Although prescription habits have since improved, the mistakes of the past continue to haunt us [2]. Opioid-related overdoses continue to rise, with an increasing number now related to heroin and illicitly manufactured fentanyl and fentanyl analogues [3]. Alarmingly, it has been shown that 75% of heroin users report that their first opioid was a prescription drug [4]. In 2019, there were 70,630 drug overdose deaths in the United States (21.6 deaths per million population), and over 70% of those deaths involved opioids [5]. While the European Union has fewer drug-induced deaths overall (14.8 deaths per million in the population aged 15 to 64 years), most of these deaths are also found to be driven by opioids—76% of recorded cases [6]. The economic burden of opioid use disorder (OUD) is immense, estimated to be approximately US $1.02 trillion in 2017 when taking into account fatal opioid overdoses, increased health care costs, and loss of productivity [7]. It is estimated that, for patients with OUD, health care costs are 8.7 times higher than for the average person. They are also 4 times more likely to end up in the emergency department and 12 times more likely to be admitted to hospital [8]. OUD and opioid dependence also affect workplaces. People with OUD do not show up for work for an average of 2.2 days per month, whereas the average person only misses an average of 0.8 days per month [8].

More than 300 million surgical procedures are performed worldwide each year, and opioids remain a primary approach for managing postoperative acute pain [9,10]. Studies have demonstrated that a significant number of patients do not discontinue opioid treatment and continue to use opioids for months or even years after surgery [10]. The percentage of patients who develop persistent opioid use (POU) depends on the definition of POU. One study found 29 different definitions of POU, and the patients who developed it ranged from 0.01% (10 patients fulfilled the definition criteria) to 14.7% (23,442 patients fulfilled the definition criteria) [11]. An Iceland-based study conducted on patients undergoing cardiac surgery found that 4.6% developed POU. When including all patients who filled an opioid prescription after surgery, that number rose to 10.1% [12]. Another study that looked at patients undergoing hand surgery with a cohort of 49% opioid-naïve patients found that 21% developed POU [13]. Both the Icelandic and hand surgery studies defined POU as filling at least one opioid prescription in the first 90 days postoperatively and then another one between 90 and 180 days postoperatively. One study also included patients who filled a prescription in the perioperative phase as part of their POU definition [12,13].

### Opioid Management

Tapering and management of prescription opioids is a well-known practice and is a part of the current clinical guidelines on safe prescribing. Every patient should receive thorough monitoring, education, and a tapering plan when prescribed opioids or receiving refills after a prolonged treatment [14-16]. However, there are challenges associated with tapering. Creating tapering plans is a time-consuming process in which clinicians can spend 15 minutes or more on each plan. To contextualize this, take, for example, a general practitioner making up to 3 tapering plans a day. With time pressure and normalization of working overtime, reducing this to 2 minutes per plan generated could save the practitioner up to 40 minutes of work per day, adding up to 3.3 hours of work over the week. Given the time-consuming nature of the current method of developing tapering plans, one can see how this could influence a clinician’s likelihood of even offering tapering to patients. This is especially relevant to high-volume prescribers. This increased efficiency may encourage wider implementation of tapering support in routine care.

Studies have shown that most medical doctors are so heavily burdened with documentation through electronic health record work and desk tasks that they cannot manage their daily duties [17]. Patients can develop physical tolerance to opioids quickly, which results in withdrawal syndrome if doses are not tapered; this, in turn, often prolongs the treatment [14,16]. After just a few weeks of opioid use, dependence can develop, where patients begin to rely on the medication not for its pain-relieving effects but due to an emerging addiction. Often, they are unaware of the dangerous progression they are facing. This can lead to the development of OUD [14]. When prescribing opioids after surgery, the total duration of opioid use is the strongest predictor of misuse. Each refill is associated with a 44% increase in the rate of misuse, and each additional week of use increases the risk of misuse by 20% [18]. According to the World Health Organization and the United Nations Office on Drugs and Crime, the largest risk factor regarding misuse of addictive drugs is the lack of education and understanding of their adverse effects. Other risk factors include easy access, naive social attitude toward these drugs, and poor prescribing habits [19,20]. Women are at higher risk of developing opioid addiction. On the other hand, men are much more likely to be prescribed opioids and represent 75% of opioid-related deaths [8,21]. However, there are also factors that are associated with opioid tapering success. One study shows that some risk factors for developing opioid dependence and abuse are also associated with opioid tapering success. Comorbidities such as substance use disorder and anxiety, along with other notable factors such as young age (21-49 years), surgery in the previous year, and nonnarcotic analgesic medication use, increased the odds of patients successfully lowering their opioid dose by 50% in 1 year. There is limited information on this topic, but the available data present intriguing implications [22].

### The Future of Follow-Up?

The evolution of follow-up through smartphone apps has shown promising results in other fields of medicine, such as cardiac rehabilitation and diabetes [23,24]. Furthermore, patients are increasingly interested in the idea of using smartphone apps for follow-up, and the recent COVID-19 pandemic has dramatically increased the use and acceptability of digital health solutions [25]. A review from 2020 looked at both the effectiveness and cost efficiency of telemedicine tools for various diseases and health problems [26]. In total, 40 of 53 reviews included in this umbrella review focused on effectiveness and used device- or app-based monitoring of patients, similar to what we propose in this protocol. There were 18 reviews focused on cost-effectiveness [26]. Most reported either (1) that the intervention was cost-effective or (2) that evidence was limited but pointed toward cost-effectiveness [26]. This is not enough to draw conclusions as the reviews were from different specialties of medicine. Just the possibility of lower costs regarding in-person meetings and hospitalizations, reduction in stigma, and a higher level of support emphasizes the justification for testing this intervention. It has also been found that telemedicine is superior or at least comparable to face-to-face communication in targeting lifestyle factors such as alcohol consumption, medication adherence, physical activity, and sleep habits [27-31]. The proposed project aims to address the challenge of reducing long-term opioid use after surgery, which is extremely important given the substantial volume of surgical procedures conducted worldwide.

## Methods

### Design

This protocol describes a prospective exploratory cohort study. The usual care procedure entails discharging patients after surgery with an opioid prescription, verbal instructions on how to taper opioid medication, and a printout of the tapering instructions upon request. The experimental procedures in this study are the referral to the Prescriby clinic alongside treatment and follow-up offered by a clinical pharmacist using the Prescriby application.

The primary target population for this study are patients undergoing surgery; surgeons; and other health care professionals involved in opioid prescription, management, and tapering. The patients are the main participants in this study and have a platform to voice their concerns or satisfaction in an exit interview at the conclusion of the intervention. The satisfaction of patients will be measured using a questionnaire at the intervention’s conclusion. The surgeons and other health care professionals connected to the patients and the processes involved have the chance to contact Prescriby directly with their concerns or to express satisfaction.

### Outcomes

The primary and secondary research questions that will be investigated are presented in Textbox 1.

**Primary and secondary research questions.**

**Primary research questions**

- Do patients who completely tapered off opioids successfully sustain tapering of the opioid medication at 6 and 12 months after the intervention?
- Do the patients who partially tapered off opioids maintain their reduced dose at 6 and 12 months after the intervention?

**Secondary research questions**

- How many successful tapers off opioids are achieved with the intervention?
- How many participants reduce their opioid dose with the intervention?
- What percentage of users are active users (ie, those who completed at least 80% of data registrations during the intervention and underwent both the introduction and exit interviews)?
- Are the participants satisfied with the intervention?
- Do patients experience increased pain symptoms during the intervention?
- Do patients experience opioid withdrawal symptoms during the intervention?

### Participants

All participants enrolled in this study have been referred to the Prescriby clinic from orthopedic clinics and the orthopedics department at Landspítali University Hospital after elective knee or hip replacement surgery. They must be prescribed opioids after surgery, own a smartphone, and be over the age of 18 years to be included in this study.

The exclusion criteria are (1) recent history of nonoral self-administration of narcotics, (2) recent history of attempted suicide, and (3) untreated mental illness.

The exclusion criteria are the same as for the Prescriby clinic services in general. The clinic not only serves the orthopedics cohort but also offers services through primary health care for long-term users of benzodiazepines, opioids, and Z-drugs. We do not exclude oral or smoked illicit opioid users from our services but require these patients to receive care at the outpatient ward of the National Center of Addiction Medicine for assessment of their treatment options.

The criteria to stop the intervention are as follows: (1) 3 or more dose increases or continuations of the same dose, (2) the patient wanting to stop participation, (3) the patient being admitted to hospital, (4) the patient having to undergo another surgery, (5) the patient’s adverse effects being deemed too serious through a clinician assessment, (6) the patient showing threatening behavior toward the clinician, and (7) an untreated medical condition (eg, endometriosis or mental illness) arising that contributes to pain and needs to be resolved before tapering. Justification for these criteria is the safety of the participants and the clinician.

Most of the criteria for stopping the intervention are connected to escalation of care or the transfer of care to other health care workers, where the responsibility for medication management is not in the hands of clinicians at the Prescriby clinic. Exceptions to this are if the patient wants to stop tapering treatment, in which case the patient can decide what type of health care to accept, or if they show threatening behavior toward the clinician, in which case the safety of the clinician is a priority.

There will not be any sampling; all patients who consent to participation will be included. The estimated number of participants will be between 150 and 200.

A comparison group will be included in the study. This group will be composed of all patients who undergo hip or knee replacement surgery in Iceland and who receive usual care during the same period as the intervention (eg, from approximately November 2024 to April 2025). The following surgery codes of the Icelandic version of the Nordic Medico-Statistical Committee Classification of Surgical Procedures will be used to identify this group: NFB (primary prosthetic replacement of hip joint) and NGB (primary prosthetic replacement of knee joint). The personal identification numbers from the intervention group will be removed from the comparison group.

### Setting and Intervention

The intervention itself is the use of the Prescriby tapering software alongside clinician follow-up. A more detailed description of the clinic setting and the mobile phone app follow-up is provided in the following sections.

#### Prescriby Clinic and Service

The Prescriby clinic opened in collaboration with the National Center of Addiction Medicine and has 2 clinical pharmacists and a physician, hereafter referred to as the clinicians. The clinic serves different patient groups, one of which is patients who are prescribed postsurgical opioids.

The clinic receives its patients from a safe web-based referral form that has been distributed to Icelandic health care workers (eg, in orthopedics departments). A patient from the orthopedics department is referred to the clinic on the day they are discharged from the ward with their opioid prescription. They receive the same education as other patients and receive information on the starting dose of opioids to take at home. They are offered information on the Prescriby clinic and the study. The day after discharge, the patient undergoes an introduction interview over the phone with the Prescriby clinic. From this point on, the clinic handles prescription responsibility. The Prescriby clinician will offer information on the study, the terms of service, and the treatment and obtain consent using the relevant consent form (supplementary file available upon request). After this, the patient is onboarded to the Prescriby app, and follow-ups are scheduled. Before onboarding patients, the Prescriby clinicians receive training in the use of the Prescriby app, communication, and deprescribing care and follow-up of patients. The participants are offered support in the form of a phone number if they wish to contact a study investigator whom they can ask questions or obtain further information from about what they are consenting to. During onboarding, participants can optionally enter baseline characteristics such as age and gender. The clinician and patient talk about the tapering plan and implement a personalized one. Specifics on the tapering are input into the system, which includes medication, starting dose, and tapering treatment length. The system then generates a plan with a roughly even dose reduction corresponding to the available medication strength in Iceland. The clinician can then change the plan to their and their patient’s desires before the plan is set up or at any point during the tapering progress. Finally, the clinician offers the patient a chance to ask questions about the service or the introduction interview. During the course of treatment, the clinician monitors the information input by the patient into the mobile phone app bidaily and adjusts follow-up if necessary. When a patient has finished or is close to finishing tapering services, the clinician books an exit interview with the patient.

The standard opioid tapering protocol from the orthopedics department for elective knee and hip replacement surgery is approximately 2 weeks, but this can be shorter or longer if needed. Most patients are started on 5-mg long-acting oxycodone tablets and are prescribed 5 to 10 mg twice daily. The standard care offered currently includes this prescription and an interview 6 to 8 weeks after surgery. Standard care is patient initiated, so if there is a problem (eg, severe pain or prescription problems), the patient has to contact the orthopedics or emergency department or primary care to solve it. The intervention described previously is more clinician initiated as the clinician monitors the data twice daily and simplifies the process after discharge from the orthopedics department and the clinic oversees the prescription for the opioid medication.

#### The Prescriby Application

**Figure 1:**
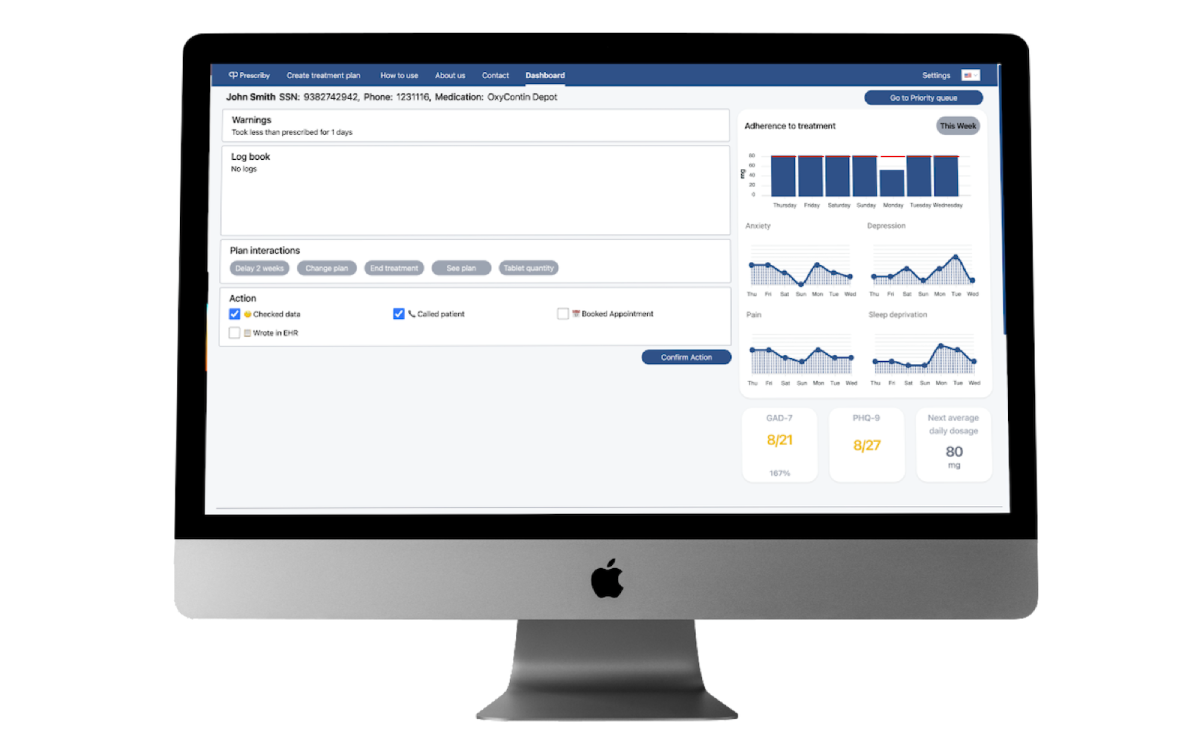
Clinician-facing module of the Prescriby application on the web browser.

**Figure 2:**
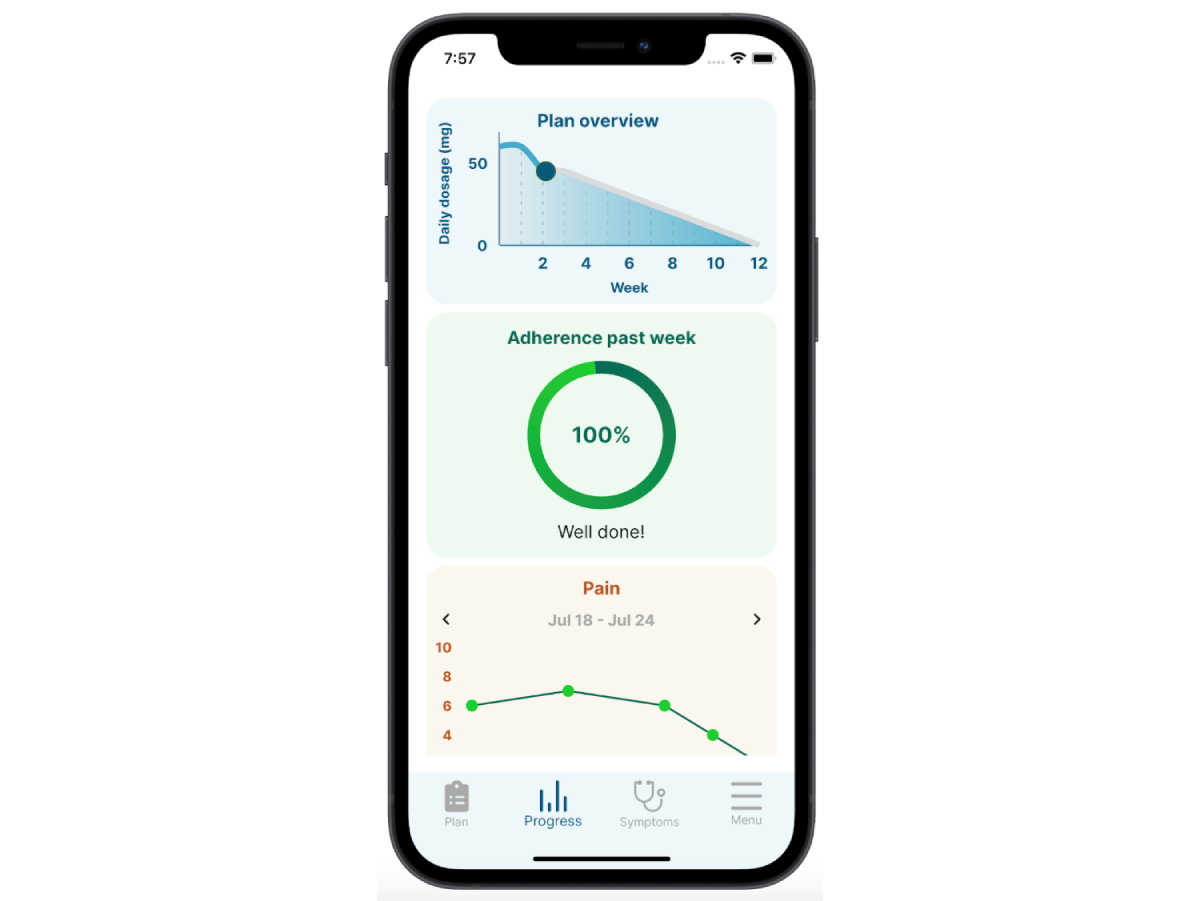
Patient-facing module of the Prescriby application on the mobile app.

The Prescriby application is designed to support patients when tapering off medication that can lead to dependence and addiction. It has 2 modules: the dashboard for the clinician and the mobile app for the patients. Images of these 2 modules can be found in Figures 1 and 2.

The clinician-facing dashboard is viewed on a computer. The clinician can make new tapering plans on the onboarding screen, see their patients on the dashboard, and set up “flags” that make the patient a higher priority in follow-up. When onboarding patients onto the app, the clinician creates the patient profile using the patient’s social security number, phone number, and name. During onboarding, the clinician must set up a plan that can be adjusted later. The clinician can also send questionnaires to patients from the dashboard to the app that the patient will be prompted to fill out the next time they open the app.

The patient-facing app is used on a smartphone. The patient-facing app has an interface where the patient can register their adherence to the tapering plan and an interface where the patient can see the data that they have input. It also has a questionnaire section where the questionnaires that the clinician has opened up can be accessed to log symptoms. Patients can see an overview of the tapering plan and their progress on the app.

### Collected Data and Covariates

#### Intervention Group

Data will be collected from the Prescriby application database and from the Prescription Medicines Database through the Directorate of Health of Iceland in the 6 and 12 months after surgery and intervention follow-up, which requires patient consent. All data will be encrypted to prevent personal identification. The dataset will be kept for 5 years after the last in a series of publications based on this study. This will allow the investigators to validate the results if requested. After 5 years, those data will be destroyed.

The data for the primary outcome measures will be extracted from the Prescription Medicines Database through the Directorate of Health of Iceland. The data for the secondary outcome measures will be extracted from the Prescriby database. Successful tapers are defined as instances in which the patient successfully tapers down and stops the medication. Reduction in opioid dose will be considered for those who did not stop the medication. These 2 outcomes will be assessed through a comparison from the start of the intervention to the end. Patients can optionally provide their gender and age. Participants will be asked about opioid use 3 months before surgery in the introduction interview.

The engagement metrics collected at the end of the intervention will be (1) active users (ie, those who completed at least 80% of data registrations during the intervention and underwent both the introduction and exit interviews) in numbers and percentages, (2) total active days in medians and IQRs, and (3) highly engaged users (ie, those who registered dosage at least 5 days a week) in numbers and percentages.

Experience and usability outcomes collected at the end of the intervention will be captured via the following statements: (1) “I would recommend this intervention to others,” (2) “The educational material was helpful,” (3) “The mobile application was user-friendly,” (4) “This intervention has improved my life and well-being,” and (5) “This intervention was better that the standard care currently offered” (the usual care procedure would be discharging patients after surgery with an opioid prescription, verbal instructions on how to taper opioid medication, and a printout of the taper upon request).

Answers are scored on a 7-point Likert scale ranging from 1 (“don’t agree at all”) to 7 (“very much agree”).

Data collection on subsequent opioid prescriptions will be conducted at 6 and 12 months after surgery. Data will only be collected on medication with the Anatomical Therapeutic Chemical (ATC) Classification System code N02A (opioids). Data on pain symptoms will be collected using the numeric pain rating scale for all patients, and data on opioid withdrawal symptoms will be collected using the Subjective Opiate Withdrawal Scale if needed. These data will be collected at the start of the intervention if needed, during the intervention, and at the end of the intervention.

#### Comparison Group

Data will be collected from the Prescription Medicines Database and Hospital Discharge Register through the Directorate of Health of Iceland in the 6 and 12 months after surgery. All data will be encrypted to prevent personal identification. The dataset will be kept for 5 years after the last in a series of publications based on this study if the investigators need to validate the results upon request and will then be destroyed. The comparison group will only be used to compare with the results of the primary outcomes of the intervention group, not the secondary outcomes.

The following data will be requested from the Directorate of Health of Iceland 6 and 12 months after the intervention for the comparison group: (1) age; (2) gender; (3) medication name of opioid, ATC code N02A, prescribed after surgery; (3) number of medication prescriptions with ATC code N02A down to the fifth level 6 months after surgery; (4) number of medication prescriptions with ATC code N02A down to the fifth level 12 months after surgery; (5) number of physicians who prescribe medication with ATC code N02A down to the fifth level 6 months after surgery; (6) number of physicians who prescribe medication with ATC code N02A down to the fifth level 12 months after surgery; and (7) *International Classification of Diseases* codes from Table 1.

These data on the comparison group will be gathered from the Directorate of Health of Iceland and using the surgery codes of the Icelandic version of the Nordic Medico-Statistical Committee Classification of Surgical Procedures, and all patients who have received Prescriby services will be removed from that list. From the Directorate of Health, we will obtain information on the same diagnoses found in Table 1 that we will have also collected from the intervention group. This will help us extract a comparison group with similar ages, genders, and comorbidities that affect physical function to those of our intervention group. Finally, we will also collect opioid prescription data on this comparison group and our intervention group through the Directorate of Health.

The results from the comparison group will be compared to the results from the intervention group. The results will be presented as the number of refills in that period and as the percentage of the comparison group that receives a refill. The results will be presented without personal identification.

Comparison of the baseline characteristics of age, gender, state of chronic opioid use before surgery, and comorbidities found in Table 1 and starting opioid dose will be conducted between the intervention and comparison groups. These characteristics will be shown in the proposed table on the demographics of the participants when the results are published.

**Table 1:** Comorbidities that affect physical function; scoring; and their International Classification of Diseases, 10th Revision–Clinical Modification (ICD-10-CM) codes [32,33].

Comorbidity	*ICD-10-CM* codes	Are any of the *ICD-10-CM* codes present? (Yes=1; no=0)
Arthritis (rheumatoid and osteoarthritis)	M05^a^, M06^a^, M08.0^a^, M08.2^a^, M08.3, M08.4^a^, M08.8^a^, M08.9^a^, M12.0^a^, M15^a^, M16^a^, M17^a^, M18^a^, and M19^a^	
Osteoporosis	M80^a^ and M81^a^	
Asthma	J45^a^	
Chronic respiratory disease (chronic obstructive pulmonary disease, chronic respiratory distress, or emphysema)	J43^a^, J44^a^, J47^a^, J60, J61, J62^a^, J63^a^, J64, J65, J66^a^, J67^a^, J68.4, J70.1, J96.1^a^, and J96.2^a^	
Angina	I20.1, I20.8, and I20.9	
Congestive heart failure or heart disease	I09.81, I24^a^, I25.1^a^, I25.3, I25.4^a^, I25.5, I25.6, I25.7^a^, I25.8^a^, I25.9, and I50^a^	
Myocardial infarction	I21^a^, I22^a^, and I25.2	
Neurological disease	G04.1, G10, G11^a^, G12^a^, G20, G21^a^, G25.4, G25.5, G30^a^, G31.0^a^, G31.1, G31.83, G31.84, G31.85, G31.89, G31.9, G32.81, G35, G36^a^, G37^a^, G40^a^, G80^a^, G81^a^, G82^a^, G83.0, G83.1^a^, G83.2^a^, G83.3^a^, G83.5, G83.8^a^, G83.9, and G93.1	
Stroke or transient ischemic attack	G45.0, G45.1, G45.2, G45.8, G45.9, G46.0, G46.1, G46.2, I60^a^, I61^a^, I63^a^, I67.84^a^, and I69^a^	
Peripheral vascular disease	I70.2^a^, I70.3^a^, I70.4^a^, I70.5^a^, I70.6^a^, I70.7^a^, I70.92, and I73.9	
Diabetes (type 1 or 2)	E08^a^, E09^a^, E10^a^, E11^a^, and E13^a^	
Upper gastrointestinal disease	K20^a^, K21^a^, K22.1^a^, K22.7^a^, K25^a^, K26^a^, K27^a^, K28^a^, and K29^a^	
Depression	F31^a^, F32^a^, F33^a^, F34^a^, and F39	
Anxiety or panic disorder	F40^a^, F41^a^, F42^a^, and F43.1^a^	
Visual impairment	H54^a^	
Hearing impairment	H83.3^a^, H90^a^, and H91^a^	
Back disease (degenerative disc disease, spinal stenosis, or severe chronic back pain)	M08.1, M45.0, M45.4, M45.5, M45.6, M45.7, M45.8, M45.9, M46.4^a^, M47.10, M47.14, M47.15, M47.16, M47.20, M47.24, M47.25, M47.26, M47.27, M47.28, M47.814, M47.815, M47.816, M47.817, M47.818, M47.819, M47.894, M47.895, M47.896, M47.897, M47.898, M47.899, M47.9, M48.00, M48.04, M48.05, M48.06, M48.07, M48.08, M51^a^, M54.14, M54.15, M54.16, M54.17, M54.3^a^, M54.4^a^, and M96.1	
Obesity (BMI≥30 kg/m^2^)	E66.0^a^, E66.1, E66.2, E66.8, E66.9, Z68.3^a^, and Z68.4^a^	

^a^All subclasses for the relevant *ICD-10-CM* code.

### Timelines

Definitions of the data gathered can be found in the Collected Data and Covariates section.

The data gathered are shown in Table 2. The study timeline encompasses 20 months: a recruiting period for the first 6 months, the 6-month period after surgery on the 12-month mark, 12 months after surgery on the 18-month mark, analysis and data processing during months 18 and 19, and then the write-up period during month 20.

**Table 2:** Patient-centered data gathering timeline.

		D0 (introductory interview; baseline)	D1 (follow-up and exit interview; 2-8 weeks)	D2 (6 months after surgery)	D3 (12 months after surgery)
**Intervention group**					
	Consent	✓			
	Starting dose	✓			
	Age	✓			
	Gender	✓			
	Comorbidities		✓		
	Opioid prescription data			✓	✓
**Comparison group**					
	Starting dose			✓	
	Age			✓	
	Gender			✓	
	Comorbidities			✓	
	Opioid prescription data			✓	✓

### Analysis

Descriptive statistics for background factors, age, gender, and comorbidities will be presented. Analysis of binomial variables related to the primary and secondary outcomes will be presented as a binomial proportion with Wald CIs, with the confidence level set to 95%.

Ordinal variables measured before and after the intervention will be analyzed using a paired Wilcoxon signed rank test, with significance level set at .05. An a priori power analysis was conducted for the primary dichotomous outcome assuming a 2-sided α level of .05, power of 0.80, and an anticipated prevalence of opioid prescriptions 12 months after surgery 5% to 10% in the study group compared to a presumed population incidence of 20%. To account for uncertainty in the expected incidence, the power analysis was repeated using anticipated incidences of 5% and 10%, and the larger resulting sample size was selected to ensure adequate power across a plausible range of scenarios [34,35]. A clinician involved in the intervention, data management, and follow-up will analyze the data. The plan for data management and analysis will be reviewed and validated by an external statistician. There is a possibility of publication bias, which is negated by the prospective registration of this study and a mandated publication of results regardless of whether they are positive or negative.

### Ethical Considerations

The participants are informed of this during acquisition of consent with the clinician in the introduction interview. They have access to the investigator with questions and can at any time opt out of the research. The offer their consent with an electronic ID signature and the authors attest to maintaining privacy and confidentiality of the participants’ data and identity. As this study is exploratory, requires handling of personal data, and seeks to improve existing practice, it requires the approval of a bioethics committee. The National Bioethics Committee of Iceland has given approval for a study using this protocol. This study has been given the registration number VSNb2024090010/03.01 and will be running from November 2024 to April 2026.

## Results

This study was funded in February 2024. The results of this research will be published in a peer-reviewed journal. Data collection for the secondary outcomes started in November 2024 and ended in August 2025. Data collection for the primary outcomes will start in June 2026 and finish in July 2026. As of manuscript submission in August 2025, a total of 75 patients have been recruited into the study. Analysis of secondary data started in August 2025 and is expected to end in September 2025. Results will be published in the fourth quarter of 2025. Analysis of primary data will start in July 2026 and is expected to end in September 2026, and results will be published in the fourth quarter of 2026. The authors will apply to scientific publications to publish the results, estimated in September 2025 for the secondary results and in July 2026 to September 2026 for the primary results. Preliminary results will be presented as a poster at the European Geriatric Medicine Society congress in Reykjavík on September 24 to 26, 2025. If other opportunities arise, the results might be presented at other congresses. Disseminating the results through these channels allows for wider visibility and accessibility of the findings within the local medical community.

## Discussion

### Principal Findings and Comparison to Previous Work

It is difficult to estimate exactly how many patients could develop POU in the year after elective knee or hip surgery. The percentage of POU a year after surgery ranges from 0.01% to 21% depending on the definition of POU and surgery type [10-13]. We anticipate that the POU in standard treatment (the comparison group) could be somewhere in the range of 10% to 15%. The hypothesis is that the intervention will produce a lower percentage of POU than what is observed in the comparison group. With regard to the secondary outcomes, we estimate that more than 85% of the patients in intervention group will be able to taper completely off their opioid medication and anticipate that the participants will be satisfied with the intervention.

### Strengths and Limitations

A strength of this planned study is that it will address a gap in the current scientific knowledge base. The study itself will contribute to the ever-changing eHealth scene and will offer new insights into the field of postsurgery tapering and medication monitoring. The protocol and its research questions are feasible, interesting, novel, ethical, and relevant. It is especially relevant to the current hot topic in health sciences: the deprescribing of medications. The scientific benefit of conducting this exploratory research lies primarily in obtaining better evidence on the outcomes of tapering off dependence-inducing medications. Other strengths of this research are its replicability and transparency. The authors have provided a detailed protocol describing how the intervention itself works and how to implement it. The authors have also been transparent regarding each aspect of the service offered. The authors will also distribute their dataset for validation upon request.

This study involves comparison of standard treatment (a reactive patient-driven health care service) and an intervention in the form of a mixed patient- and a clinician-driven health care service with a higher level of support than that of standard treatment. The primary outcomes rely on prescription data for both the comparison and intervention groups. This could affect the outcomes as real medication intake may be over- or underestimated. The intervention and comparison groups have an observational cohort design without random assignment. This will increase the risk of selection bias and confounding, making it difficult to attribute outcomes in tapering success or opioid use directly to the intervention.

### Future Directions

The team is looking at future research opportunities, the clinic setup, and tapering outcomes with other patient groups undergoing surgery and patients with chronic opioid use and expanding on benzodiazepine tapering. In these research opportunities, emphasis will be placed on research design that enables randomization to compare a group using the application alone as an intervention with a group with a similar level of support.

## Abbreviations

ATC: Anatomical Therapeutic Chemical
OUD: opioid use disorder
POU: persistent opioid use
